# Switch-Off response to mid-cingulate cortex stimulation: a video case report

**DOI:** 10.1016/j.cnp.2025.10.008

**Published:** 2025-11-07

**Authors:** Paola Vassallo, Olivia Poole, Xiaodong Guan, Davide Giampiccolo, Eduardo Marques, Umesh Vivekananda, John S. Duncan, Beate Diehl

**Affiliations:** a Department of Clinical & Experimental Epilepsy, UCL Queen Square Institute of Neurology, London WC1N 3BG, UK; b Chalfont Centre for Epilepsy, Chalfont St Peter SL9 0RJ, UK; c Stichting Epilepsie Instellingen Nederland (SEIN), Heemstede 2103 SW, Netherlands; d Department of Neurology, Leiden University Medical Centre, Leiden 2333 ZA, Netherlands; e National Hospital Neurology and Neurosurgery, Department of Clinical Neurophysiology, London WC1N 3BG, UK; f Victor Horsley Department of Neurosurgery, National Hospital for Neurology and Neurosurgery, London WC1N 3BG, UK; g Institute of Neurosciences, Cleveland Clinic London London SW1X 7HY, UK

**Keywords:** Electrical cortical stimulation, SEEG, Cingulate cortex

## Abstract

**Background:**

Electrical cortical stimulation (ECS) is commonly used during invasive presurgical evaluation of drug-resistant focal epilepsy for functional mapping and delineation of the epileptogenic zone.

**Case presentation:**

A 31-year-old woman underwent stereo-EEG evaluation for presurgical assessment of drug-resistant focal epilepsy, likely left frontal or multifocal. During 50 Hz ECS of the right mid-cingulate cortex, a reproducible behavioural response was observed, characterised by left ear popping, head drop, eye closure, and speech/motor arrest, without loss of awareness. This semiology differed from her habitual seizures, and the region was not considered part of the presumed epileptogenic zone.

**Conclusion:**

This case represents a rare video-documentation of stimulation-induced complex motor and behavioural response localised to the mid-cingulate cortex and supports mid-cingulate’s role as connection hub for volitional control and sensorimotor integration.

## Highlights

• Mid-cingulum cortex stimulation elicited sensory and negative motor phenomena.
• Mid-cingulum cortex stimulation triggered an unusual, complex behavioural response.
• Mid-cingulum plays a role in behavioural network and sensorimotor integration.

## 1 Introduction

Intracranial monitoring through stereoelectroencephalography (SEEG) is necessary to establish candidacy for epilepsy surgery in some people with focal drug-resistant epilepsy. Extra-operative cortical stimulation (or direct electrical cortical stimulation, ECS) is a crucial tool for functional mapping, assisting in identifying eloquent cortical areas—such as language or motor regions—and thereby minimising the risk of neurological deficits following resective surgery.

In addition to functional mapping, ECS also provides valuable insights into seizure networks and cortical connectivity, aiding in delineating the epileptogenic zone (Borchers et al., 2011).

ECS can elicit a wide range of electroclinical responses, including afterdischarges, subclinical seizures, or stimulation-induced seizures that are consistent or not with spontaneous seizures. Non-habitual seizures elicited by ECS are particularly frequent in cases of frontal or multifocal epilepsy (Spilioti et al., 2020).

We present a short video report of an unusual clinical response during ECS of the right middle cingulate cortex in a woman undergoing invasive presurgical evaluation for focal drug-resistant epilepsy.

## 2 Case description

A 31-year-old right-handed woman with drug-resistant epilepsy since the age of two presented with focal seizures associated with loss of consciousness and complex motor phenomena, such as vocal and gestural automatisms, often preceded by psychic auras. Seizures typically occurred during sleep and frequently in clusters of up to fifteen events. Her past medical history was unremarkable. Repeated high-resolution brain MRI scans revealed no evidence of potentially epileptogenic lesions. Neuropsychological testing demonstrated fronto-temporal lobe dysfunction without clear lateralising value.

**Fig. 1:**
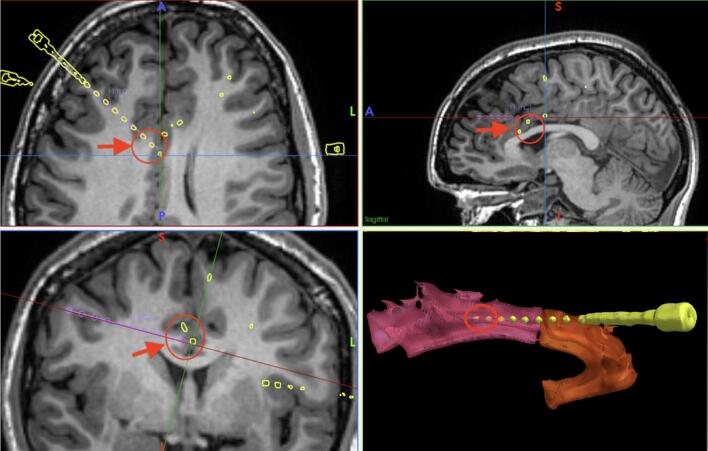
Stereo-EEG implantation plan showing the deep electrode position on MRI and the site of electrical cortical stimulation in the right mid-cingulate cortex. Contact sites encircled and indicated by the arrow: RmC1 (bottom) almost out in space below falx, RmC2 (middle) grey/white matter boundary, RmC3 (top) grey matter of middle cingulum cortex.

Prolonged scalp video EEG recordings suggested that the presumed epileptogenic zone was most likely located in the left medial frontal or cingulate region. Ictal SPECT demonstrated involvement of the left frontal lobe, particularly within the anterior cingulate cortex. However, the possibility of a right-sided or bilateral frontal epileptogenic zone could not be excluded, given the occurrence of similar semiology in seizures presumed to originate from the right or bilateral frontal regions. Therefore, SEEG electrodes were implanted bilaterally in the frontal lobes, with greater density on the left (see Fig. 1 and Legend 1). Electrode placement was confirmed through co-registration of post-implantation CT with pre-operative MRI.

The SEEG study confirmed that habitual focal seizures with loss of consciousness and complex motor features arose from the left frontal region, near the anterior/mid-cingulate electrodes. This was further corroborated by 50 Hz ECS, which reproduced habitual seizures. The study was complicated by a cluster of subclinical or focal seizures with loss of consciousness that were semiologically slightly different from the previously described and observed habitual seizures. These events had a non-localisable EEG onset, though some indicators suggested a possible origin in the right hemisphere (e.g. maximal low voltage fast activity in the right frontal region, or pre-SMA or mid-cingulate).

ECS was undertaken for mapping with the following parameters: biphasic pulses of 50 Hz, pulse width 1025 usec, starting at an intensity of 1 mA and increasing in 1 mA increments up to a maximum of 3 mA. At the time of the recording and cortical stimulation, the habitual anti-seizure medications were administered: levetiracetam 1750 mg/day, zonisamide 500 mg/day, clobazam 20 mg/day; the clobazam dose was reduced to 10 mg/day during the second ECS session.

Stimulation of the right mid-cingulate cortex consistently elicited a brief but distinctive clinical response, reproducible across three stimulations. The semiology included a popping sensation in the left ear, followed by head drop, bilateral eye closure, and—when stimulation was delivered during verbal or motor tasks (e.g. counting, lifting the arms, or moving the tongue)—speech and tongue motor arrest. This resembled a “switch-off reaction”. On direct questioning, the patient reported a strange sensation, a painful loud noise in the left ear, loss of eye control, a sensation of the head being pulled downwards, and loss of oral motor control, although she perceived herself as continuing to count.

Responses occurred reproducibly at 2.0–4.5 mA, and were accompanied in more than half of the stimulations by afterdischarges strictly localised to the electrodes implanted in the middle cingulum – within the grey matter and at the grey-white matter boundary (RmC2-3 and RmC1-2). These afterdischarges had a morphology of polyspikes, lasted up to 5.5 s and were not associated with clinical changes including heart rate or respiratory pattern. As the elicited semiology differed from her habitual seizures, the stimulated region was not considered part of the presumed epileptogenic zone.

Considering concerns regarding multifocal epilepsy, the patient was ultimately deemed unsuitable for resective epilepsy surgery.

## 3 Discussion

This case documents a rare and unusual clinical response to ECS of the mid-cingulate cortex, combining negative motor and cognitive-behavioural symptoms with altered alertness.

Functional neuroimaging and stimulation studies have demonstrated that the mid-cingulate cortex is involved in motor and cognitive processes, and is functionally connected to primary and secondary somatosensory cortices, as well as prefrontal and parietal regions—consistent with the semiology observed here(Pelliccia et al., 2022). These findings are in keeping with previous reports implicating the dorsal cingulate cortex(Souirti et al., 2014), and similar responses have also been described following limbic stimulation of the hippocampus and amygdala(Bregianni et al., 2022).

The occurrence of analogous responses following stimulation of anatomically distinct but highly interconnected structures (e.g. cingulum cortex, hippocampus, amygdala) highlights the limitations of interpreting ECS effects as strictly local. Although ECS is delivered focally, clinical phenomena may result from modulation or inhibition of downstream or interconnected areas, particularly in cases where the elicited effect is a negative motor response(Drane et al., 2021).

These findings support the hypothesis that the mid-cingulate cortex acts as a functional hub, integrating networks responsible for volition, alertness, and motor control. Nonetheless, the clinical relevance of such stimulation responses must be interpreted with caution. The recordings and stimulations were performed for clinical purposes, and the implantation has significant spatial limitations inherent in electrode coverage. A review of evoked responses to single-pulse electrical stimulation (SPES) of the middle cingulum revealed there was evidence of connectivity with the ipsilateral supplementary motor area, but with limited sampling, a comprehensive connectivity matrix cannot be estimated. Sensory cortices were not implanted; therefore, no conclusion can be drawn about connectivity between mid-cingulum and sensory areas, based on this study. Connectivity analyses, and multi-centre collaborative research to develop consensus protocols for stimulation and assessment are needed to enhance the generalisability of such findings.

## 4 Conclusion

This case illustrates an unusual and reproducible behavioural response to ECS of the mid-cingulate cortex, characterised by sensory phenomena and negative motor symptoms with impaired alertness. The findings support existing literature implicating the mid-cingulate cortex as a functional hub within broader brain networks involving volitional control and alertness. This report provides rare video documentation of a phenomenon that is typically described or depicted through still images, thereby offering added educational value.

## Ethical statement

Individual consent was obtained prior to SEEG implantation and recording as part of routine clinical care. Written consent for the use of video material was obtained after review of the video. The authors confirm that they have read the Journal’s position on ethical publication and affirm that this report complies with those guidelines.

## Declaration of Competing Interest

The authors declare that they have no known competing financial interests or personal relationships that could have appeared to influence the work reported in this paper.
